# Molecular evolution, diversity, and adaptation of foot-and-mouth disease virus serotype O in Asia

**DOI:** 10.3389/fmicb.2023.1147652

**Published:** 2023-03-09

**Authors:** Fangtao Li, Yan Li, Jianrong Ma, Ruizhi Wu, Xingqi Zou, Yebing Liu, Qizu Zhao, Yuanyuan Zhu

**Affiliations:** National/WOAH Reference Laboratory for Classical Swine Fever, China Institute of Veterinary Drug Control, Beijing, China

**Keywords:** FMDV, O/Cathay, genetic diversity, host tropisms, potential molecular mechanism

## Abstract

Foot-and-mouth disease (FMD) is highly contagious and affects the economy of many countries worldwide. Serotype O is the most prevalent and is present in many regions of Asia. Lineages O/SEA/Mya-98, O/Middle East-South Asia (ME-SA)/PanAsia, O/Cathay and O/ME-SA/Ind-2001 have been circulating in Asian countries. Low antigenic matching between O/Cathay strains and current vaccine strains makes the disease difficult to control, therefore, analyzing the molecular evolution, diversity, and host tropisms of FMDV Serotype O in Asia may be helpful. Our results indicate that Cathay, ME-SA, and SEA are the predominant topotypes of FMDV serotype O circulating in Asia in recent years. Cathay topotype FMDV evolves at a higher rate compared with ME-SA and SEA topotypes. From 2011 onwards, the genetic diversity of the Cathay topotype has increased substantially, while large reductions were found in the genetic diversity of both ME-SA and SEA topotypes, suggesting a trend that infections sustained by the Cathay topotype were becoming a more severe epidemic in recent years. Analyzing the distributions of host species through time in the dataset, we found that the O/Cathay topotype was characterized by a highly swine-adapted tropism in contrast with a distinct host preference for O/ME-SA. The O/SEA topotype strains identified in Asia were isolated mainly from cattle until 2010. It is worth noting that there may be a fine-tuned tropism of the SEA topotype viruses for host species. To further explore the potential molecular mechanism of host tropism divergence, we analyzed the distribution of structure variations on the whole genome. Our findings suggest that deletions in the PK region may reflect a common pattern of altering the host range of serotype O FMDVs. In addition, the divergence of host tropism may be due to accumulated structural variations across the viral genome, rather than a single indel mutation.

## 1. Introduction

FMD is a contagious viral disease clinically characterized by lesions in the mouth and feet of cloven-hoofed animals, which has affected more than 70 animal species including cattle, pigs, sheep, goats, water buffalo, and wild ruminants (Garcia, 2012; Li et al., 2021), and imposes burden on the economy of many countries worldwide (Sinkala et al., 2014; Diaz-San Segundo et al., 2017). FMD virus (FMDV) is a member of the Aphthovirus genus within the Picornaviridae family. It is ~30 nm in diameter and forms an icosahedral structure with a sedimentation coefficient of 146S, and consists of 60 copies of each of the capsid proteins VP1–VP4 (Knowles and Samuel, 2003). The capsid protein precursor (P1 + 2A) of picornavirus is initially digested into VP0, VP1, and VP3 to form a protomer. Mature virions are ultimately formed with the package of the genome into capsids and the autocatalytic cleavage of VP0 into VP2 and VP4 (Freimanis et al., 2016).

FMDV has seven antigenically distinct serotypes, namely, O, A, C, Asia 1, Southern African Territories (SAT) 1, SAT 2, SAT 3 and numerous subtypes (Doel, 2003). FMDV serotype O is one of the global epidemic serotypes and causes significant economic loss (Shao et al., 2011). Three serotypes of FMDV, including serotypes O, A, and Asia 1, have caused epidemics in Asia, making FMD difficult to control (Brito et al., 2017; Blacksell et al., 2019). The serotype O is the most prevalent of the three serotypes and is present in many regions in Asia (Zhu et al., 2019). In India, FMDV serotype O dominated the outbreak scenario, accounting for about 92% of all outbreaks (Subramaniam et al., 2022). Serotypes O is also the most prevalent in Bangladesh, and the maximum outbreaks occurred with this serotype (Hossen et al., 2020). Despite the efforts of the National FMD Control Program, A large majority of the reported outbreaks caused by FMDV serotype O in Nepal remains a major threat to the livestock industry in Nepal (Adhikari et al., 2018). Along with the three usual strains O/SEA/Mya-98, O/Middle East-South Asia (ME-SA)/PanAsia, and O/Cathay, an emerging O/ME-SA/Ind-2001 (ME-SA) lineage has been circulating in this region since 2015 and have now spread to most of the Southeast Asian countries (Upadhyaya et al., 2021). In recent years, serotype O FMDVs have been mainly responsible for outbreaks of FMD in China (Li et al., 2022).

In 1997, a FMDV confirmed in Taiwan showed atypical pathogenicity with high morbidity and mortality in swine but no effect on cattle, leading to severe economic losses (Dunn and Donaldson, 1997). The causative agent was confirmed to be a distinct topotype of serotype O (i.e., O/Cathay), which was identified for the first time in 1970 in China (Beard and Mason, 2000). Since the catastrophic outbreak in Taiwan, sporadic outbreaks caused by O/Cathay strains have been reported in China and several Southeast Asian countries, together with O/SEA/Mya-98, O/ME-SA/PanAsia, and O/ME-SA/Ind-2001 strains (Brito et al., 2017).

Although vaccination is the key to control serotype O FMD, the available vaccines are not able to provide enough cross-protection as outbreaks still occurred despite repeated vaccinations (Mahapatra et al., 2017; Lee et al., 2020; Park et al., 2021). The vaccines used showed a good match with the O/SEA and O/ME-SA viruses, whereas none of the recently circulating O/Cathay viruses were protected by any of the vaccine strains, including the existing O/Cathay vaccine, indicating an antigenic drift and the urgency to develop new vaccine strains (Upadhyaya et al., 2021). Low antigenic matching between the O/Cathay strains and current vaccine strain makes the disease difficult to control, so current strategies to eradicate FMDV of this topotype rely on the rapid detection of infected animals and control measures including movement restriction and culling of animals suspected of infection (Nishi et al., 2021). Therefore, it is necessary to analyze the molecular evolution and host tropisms of FMDV serotype O in Asia.

This study investigated the molecular epidemiology, evolutionary dynamics, and host adaptation of FMDV serotype O circulating in Asia. We found that the O/Cathay FMDV topotype evolves at a higher rate compared to other predominant topotypes in Asia. Genetic diversity of the O/Cathay topotype was estimated to increase in recent years, reflecting its elevated prevalence in this region. Differential host tropisms revealed the evolutionary divergence between O/Cathay and other topotypes. These findings suggest that O/Cathay FMDVs pose serious implications for the control of FMD.

## 2. Methods

### 2.1. Sequence data

Genome sequences and associated metadata of serotype O FMDV from Asia countries were collated from the GenBank database (Benson et al., 2013). Nucleotide sequences were aligned using MAFFT v7.505 (Katoh and Standley, 2013) and the VP1 protein-coding regions were extracted manually. The topotypes of all sequences were determined based on the phylogenetic proximity to reference topotype *VP1* sequences, using a neighbor-joining tree topology (Saitou and Nei, 1987) as implemented in the MEGA 11 (Tamura et al., 2021).

### 2.2. Phylodynamic reconstructions

The temporal signal of sequence data was examined in TempEst v1.5.3(Rambaut et al., 2016), using a root-to-tip regression of genetic distances against sampling time computed from the maximum-likelihood (ML) phylogenetic tree. The ML tree was inferred in RaxML v8.2.12 (Stamatakis, 2014) using 1,000 bootstrap replicates under GTR substitution model with gamma-discretized among-site rate variation, which was determined as the best-fitting nucleotide substitution model by ModelFinder (Kalyaanamoorthy et al., 2017).

Time-scaled phylogenies were reconstructed in BEAST v1.10.4(Suchard et al., 2018). The evolution of FMDV was modeled by parameterizing the process of nucleotide substitution using the GTR-gamma_4_ model, by allowing evolutionary rates to vary across branches according to a lognormal distributed relaxed molecular clock (Drummond et al., 2006), and by using the nonparametric Skygrid coalescent demographic model (Gill et al., 2013) as tree prior, setting 100 transition-points for population size changes. The joint posterior estimates were obtained running a Markov chain Monte Carlo (MCMC) for 100–200 million iterations, 10% of which were removed as burn-in. Mixing and convergence of the MCMC chains were then assessed using Tracer v1.7.2(Rambaut et al., 2018), to ensure sufficient sampling was achieved.

### 2.3. Structural variation identification

Genome sequences of FMDV were collected and aligned pairwise to obtain mutation information. Sequences with more than 80 ‘N’ or merged nucleotides were discarded. Structural variation information of each sequence was then extracted using a Perl script. To avoid interference from sequencing quality, only sites with a gap against normal bases (i.e., A, T, C, and G) were treated as insertions or deletions.

## 3. Results

### 3.1. Cathay, ME-SA, and SEA are the predominant topotypes of FMDV serotype O circulating in Asia

**Figure 1:**
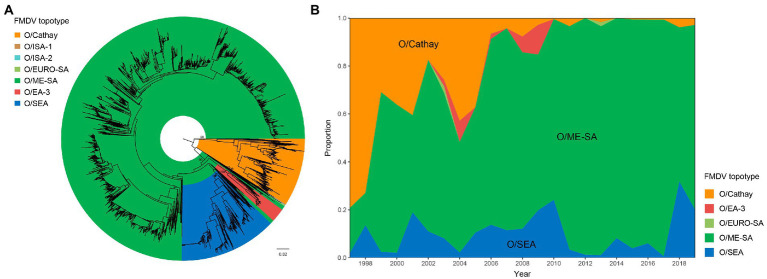
Molecular epidemiology of FMDV serotype O circulating in Asia. **(A)** Midpoint-rooted neighbor-joining tree based on VP1 gene sequences of FMDV serotype O in Asia. Node labels represent bootstrap values. Scale bar is in units of nucleotide substitutions per site. **(B)** Proportion of topotypes of FMDV serotype O in Asia between 1997 and 2019.

To investigate the molecular epidemiology of FMDV serotype O in Asia, we compiled an extensive data set of FMDV VP1-coding sequences (*n* = 3,498) and performed phylogenetic analysis using these *VP1* gene sequences. As shown in Figure 1, at least 7 topotypes of FMDV serotype O have been found in Asia so far, comprising of Cathay, Middle East-South Asia (ME-SA), Southeast Asia (SEA), Indonesia-1, Indonesia-2, Europe-South America, and East Africa 3 (Figure 1A). Among these topotypes, Cathay, ME-SA, and SEA were the three most persistent topotypes in recent decades, with the most significant number of virus isolates. In contrast, other topotypes were only detected sporadically. Furthermore, we found that multiple topotypes were co-circulating in Asia, suggestive of complex dynamics of coexisting viral topotypes evolving within and between distinct ecological systems (Figure 1B). These findings indicate that Cathay, ME-SA, and SEA are the predominant topotypes of FMDV serotype O circulating in Asia in recent years.

### 3.2. Increased genetic diversity of Cathay topotype FMDV reflects its elevated prevalence in recent years

**Figure 2:**
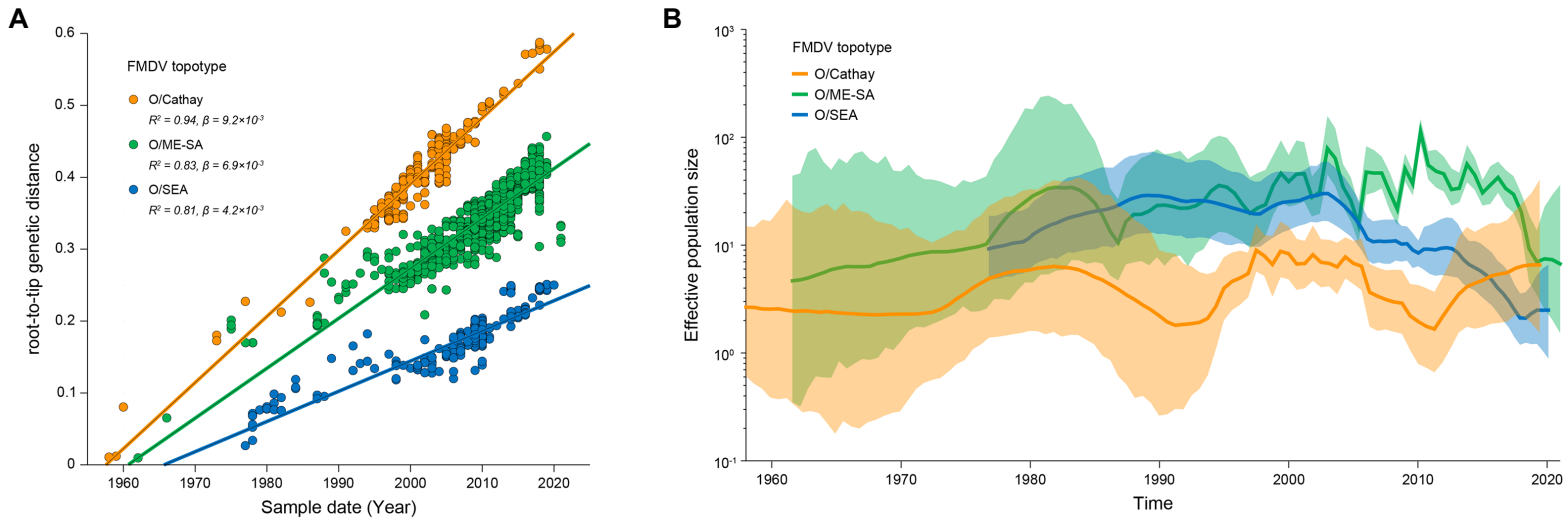
Evolutionary dynamics of dominant topotypes of serotype O FMDV circulating in Asia. **(A)** Linear regression of sampling time against divergence from the root of reconstructed maximum-likelihood trees from FMDV VP1 sequences. Circles representing tip nodes are colored according to the corresponding FMDV topotype. R-squared (*R*^2^) and slope (*β*) parameters estimated for each fitted regression line are reported. **(B)** Historical trend of genetic diversity in dominant topotypes of serotype O FMDV circulating in Asia. Lines represent median estimates of the effective population size with colored areas defining the 95% highest posterior density region.

*VP1* sequences of Cathay, ME-SA and SEA topotype FMDV were further analyzed to reconstruct the evolutionary dynamics of these prevalent topotypes in Asia. Analyses of the root-to-tip divergence estimated from the maximum-likelihood tree as a function of the sampling time revealed strong temporal signals of both Cathay, ME-SA, and SEA FMDV evolution (*R*^2^ of 0.94, 0.83, and 0.81, respectively; Figure 2A). The evolution rates were estimated to be 1.06 × 10^−2^, 0.89 × 10^−2^, and 0.68 × 10^−2^ nucleotide substitutions/site/year for Cathay, ME-SA, and SEA topotype, respectively. These results indicate that Cathay topotype FMDV in Asia evolves at a higher rate, compared with ME-SA and SEA topotypes.

The dynamics of genetic diversity of the FMDV populations can reflect fluctuations in the size of the host population through time. We further quantified the genetic diversity of FMDV among Cathay, ME-SA, and SEA topotypes. Historical changes in viral diversity before 2006 revealed a trend of cyclical dynamics of alternating topotypes (Figure 2B). It is worth noting that, from 2011 onwards, the genetic diversity of the Cathay topotype has increased substantially, while large reductions were found in genetic diversity of both ME-SA and SEA topotypes in recent years. These results would suggest a trend that infections sustained by Cathay topotype FMDV were becoming a more severe epidemic in recent years in Asia.

### 3.3. Differential host tropisms revealed the evolutionary divergence between topotypes of serotype O FMDV in Asia

**Figure 3:**
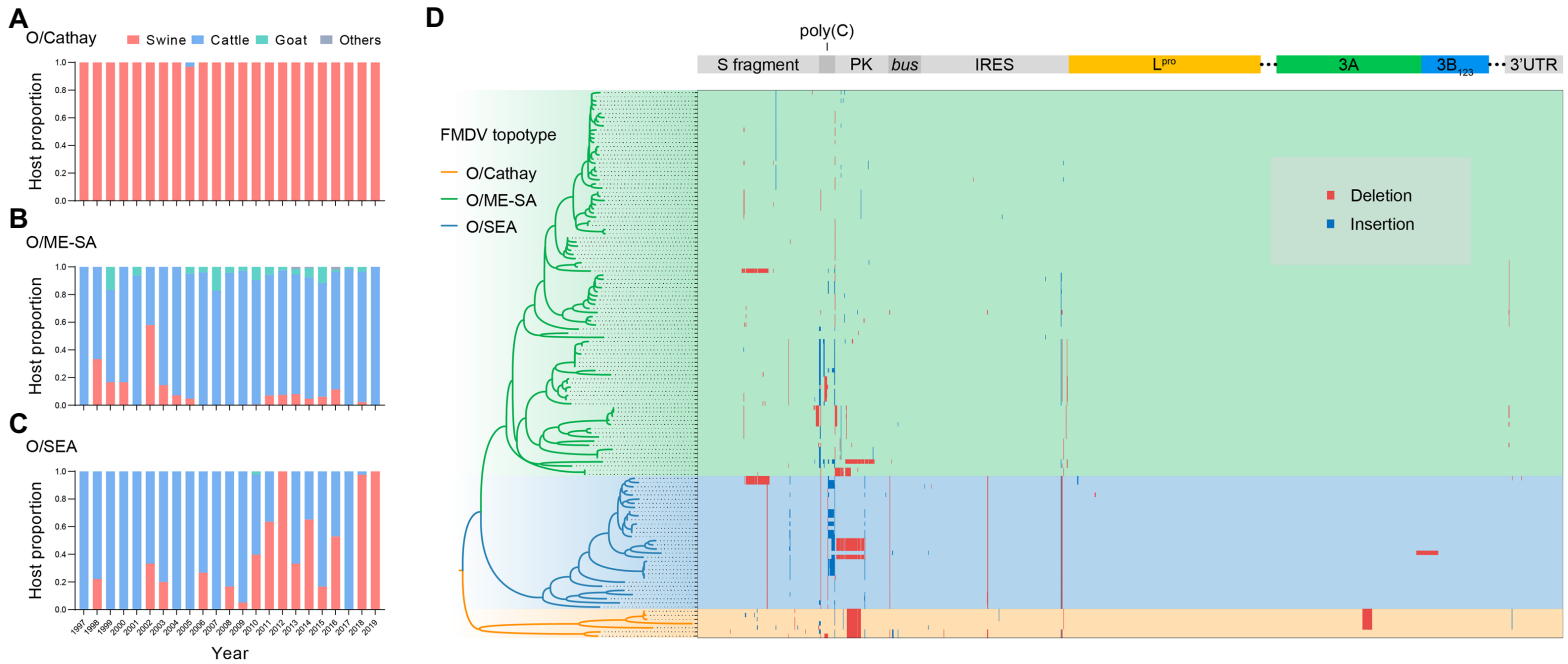
Adaptive evolution of dominant topotypes of FMDV serotype O circulating in Asia. **(A–C)**, FMDV serotype O host makeup for Cathay **(A)** ME-SA **(B)** and SEA **(C)** topotype through time. **(D)** Genomic distribution of insertions (blue) and deletions (red) in serotype O FMDV strains isolated in Asia.

Analyzing the distributions of host species through time in the dataset, we found that the O/Cathay topotype was characterized by a highly swine-adapted tropism, as 97.9% (322/329) of O/Cathay strains were isolated from swine, with the remaining 2.1% (7/329) isolated from cattle (Figure 3A). In contrast, although the O/ME-SA topotype was able to infect many cloven-hoofed animals, including swine, cattle, and sheep, the majority of O/ME-SA isolates were obtained from cattle (88.2%), indicating a distinct host preference for O/ME-SA, compared with O/Cathay topotype (Figure 3B). The O/SEA topotype strains identified in Asia were isolated mainly from cattle until 2010, after which more isolates of the O/SEA topotype were obtained from swine, not cattle. This suggests there may be a fine-tuned tropism of the SEA topotype viruses for host species (Figure 3C).

Structural variations in the viral genome, including insertions and deletions, have a greater impact relative to nucleotide substitutions on both gene structures and protein functions, facilitating better adaptation of FMDV to hosts and/or environmental conditions. To further explore the potential molecular mechanism of host tropism divergence, we systematically analyzed structural variation events in whole genomes of Cathay, ME-SA, and SEA topotype FMDVs. Across the entire genome of these three topotypes, insertions and deletions occurred in the 5′ untranslated region (UTR), nonstructural proteins, and 3′ UTR (Figure 3D). It is interesting to note the deletion events observed in the pseudoknot (PK) region. A deletion of 43 nucleotides in the PK region occurred consistently in Cathay strains, and a ME-SA strain isolated from swine had a PK region deletion of 86 nucleotides. These deletions have been shown to significantly attenuate the ability to infect cattle, with no major effects on the pathogenicity in swine (Zhu et al., 2019). Furthermore, some SEA topotype strains isolated in swine in Vietnam and South Korea also included an 86-nucleotide deletion within the PK region. However, none of the genome sequences of bovine-derived SEA strains harbored this PK region deletion. These findings suggest that deletions in the PK region may reflect a common pattern of altering the host range of serotype O FMDVs.

In the 3A protein of FMDV, a 10-amino-acid deletion has been proven to be responsible for the porcinophilic properties of FMDV in previous studies (Knowles et al., 2001). This deletion was detected in recent isolates of the Cathay topotype, but not in early isolates of this topotype or isolates of the other two topotypes (Figure 3D). In addition, a dual structural variation, a 70-nucleotide deletion in the S fragment combined with a 1-amino-acid insertion in the leader protein (L^pro^), which was demonstrated as a determinant of attenuated virulence of serotype O FMDVs in cattle (Yang et al., 2020), was observed in two SEA topotype strains isolated from swine (Figure 3D). These facts indicate that the divergence of host tropism may be due to accumulated structural variations across the viral genome, rather than a single indel mutation.

## 4. Discussion

In this study, we describe the phylogeny and evolution of serotype O FMDV in Asia. Our findings indicate that Cathay, ME-SA, and SEA are the predominant topotypes of FMDV serotype O circulating in Asia in recent years, and suggest a trend that infections sustained by Cathay topotype FMDV have become dominant in recent years in Asia. Analyses of evolutionary divergence between topotypes of serotype O FMDV highlighted the significant role of accumulated structural variations across the viral genome in the divergence of host tropism.

FMD is a global disease, which poses a major threat to the animal industry and causes enormous economic losses (Porphyre et al., 2018). Among FMDV serotypes, serotype O is most prevalent and the maximum outbreaks occurred with this serotype in Asian countries (Hossen et al., 2020). In recent years, Cathay, ME-SA, and SEA are the predominant topotypes of FMDV serotype O circulating in Asia. So far, cattle infected with FMDV of the O/Cathay topotype have a low risk of viral transmission or persistence, which is a major reason for the smaller number of outbreaks caused by this topotype compared to others (Nishi et al., 2021). However, sporadic outbreaks continue to be reported in several Southeast Asian countries. In previous studies, where and how viruses of this topotype are maintained or spread remain unclear (Di Nardo et al., 2014; Brito et al., 2017). In our research, we show that the genetic diversity of the Cathay topotype has increased substantially characterized by a highly swine-adapted tropism, causing continuous prevalence of O/Cathay. Because of low antigenic matching between the O/Cathay strain and current vaccine strains according to quarterly reports from the World Reference Laboratory for FMD^1^, strategies to eradicate FMDV of O/Cathay would rely on movement restriction and culling of animals suspected of infection (Nishi et al., 2021). The pig industry is one of the most important sectors of agriculture in most countries of Asia, where such strategies can lead to severe economic losses. Further statistical surveillance should be targeted toward O/Cathay, a kind of porcinophillic FMDV, to strategize appropriate risk management and to reduce the possibility of virus transmission.

The genetic variations in Cathay topotype viruses have accumulated over several decades. In previous research, the deletion of 43 nt in the PK region, the 10-amino-acid deletion in the 3A protein and the 70-nt deletion in the S fragment or the single leucine insertion in L^pro^ of serotype O FMDV may show a swine-adapted characteristic, resulting in the altered host tropism of the virus in cattle (Knowles et al., 2001; Zhu et al., 2019; Yang et al., 2020). Our findings in this study further indicated the important role of PK region deletion in the variation and the accumulated structural variations across the viral genome for the host tropism of serotype O FMDVs, which might be the critical determinants of viral tropism of serotype O FMDV from cattle to swine. The concurrence of these mutations in serotype O FMDV may result in the altered host range of the virus that enabled swine to become the main epidemiological host.

The serotype O FMDV is the most prevalent serotype in Asia. Asia possesses a dense pig population, which probably caused the more frequent propagation and prevalence of the swine-origin serotype O FMDV. Besides, the decreased viral pathogenicity of swine-adapted FMDV may benefit virus maintenance in the pigs, because high pathogenicity may leave the host unable to further support viral maintenance or reproduction (Ebert and Bull, 2003; Dortmans et al., 2010; Wang et al., 2012). Thus, continual systematic surveillance and more detailed investigation of prevailing serotype O FMDVs in swine populations, especially the O/Cathay topotype, are urgently needed to formulate an efficient FMD control strategy for Asia.

## Data availability statement

The datasets presented in this study can be found in the GenBank database (https://www.ncbi.nlm.nih.gov/genbank/). The accession numbers can be found in the supplementary material.

## Author contributions

FL, QZ, and YZ contributed to the conception and design of the study. YLi and JM organized the database. FL and RW performed the statistical analysis. FL wrote the first draft of the manuscript. YLiu and XZ wrote sections of the manuscript. All authors contributed to the manuscript revision, read, and approved the submitted version.

## Funding

This work was supported by grants from the National Key R&D Program of China (2021YFD1800300) and National Natural Science Foundation of China (32202791).

## Conflict of interest

The authors declare that the research was conducted in the absence of any commercial or financial relationships that could be construed as a potential conflict of interest.

## Publisher’s note

All claims expressed in this article are solely those of the authors and do not necessarily represent those of their affiliated organizations, or those of the publisher, the editors and the reviewers. Any product that may be evaluated in this article, or claim that may be made by its manufacturer, is not guaranteed or endorsed by the publisher.

## Footnotes

^1^ https://www.wrlfmd.org/ref-lab-reports
