# Supplementary material for: Molecular evolution, diversity, and adaptation of foot-and-mouth disease virus serotype O in Asia
Source: Front Microbiol. 2023 Mar 9;14:1147652. doi: 10.3389/fmicb.2023.1147652 (PMC10034406; doi:10.3389/fmicb.2023.1147652)
Supplement: Supplementary file 1 [file Table_1.pdf]

TABLE S1 Accession numbers of FMDV sequences used in this study.

Accession
AB050978, AB079061, AB618503, AF026168, AF095863, AF095864, AF095865, AF095866, AF095867, AF095868, AF095869, AF095870, AF095871, AF095872,
AF095873, AF095874, AF095875, AF095876, AF095877, AF095878, AF095879, AF095880, AF095881, AF095882, AF095883, AF095884, AF095885, AF204276,
AF292107, AF506822, AF511039, AH012984, AH012985, AJ131468, AJ131469, AJ131470, AJ251477, AJ294910, AJ294912, AJ294913, AJ294916, AJ294917,
AJ294918, AJ294921, AJ294926, AJ294927, AJ294930, AJ296328, AJ303499, AJ303500, AJ303501, AJ303502, AJ303503, AJ303509, AJ303531, AJ318824,
AJ318825, AJ318830, AJ318831, AJ318832, AJ318833, AJ318840, AJ318844, AJ318847, AJ318851, AJ318854, AJ318856, AJ539136, AJ539137, AJ539138,
AJ539139, AY114146, AY145897, AY333431, AY373583, AY593811, AY593812, AY593813, AY593823, AY593824, AY593828, AY593833, AY593834, AY593835,
DQ119643, DQ164862, DQ164863, DQ164866, DQ164867, DQ164869, DQ164870, DQ164881, DQ164882, DQ164883, DQ164884, DQ164885, DQ164886, DQ164887,
DQ164888, DQ164889, DQ164892, DQ164895, DQ164897, DQ164899, DQ164900, DQ164901, DQ164902, DQ164904, DQ164905, DQ164906, DQ164907, DQ164911,
DQ164915, DQ164916, DQ164919, DQ164920, DQ164921, DQ164922, DQ164923, DQ164925, DQ164927, DQ164928, DQ164929, DQ164930, DQ164931, DQ164932,
DQ164933, DQ164934, DQ164935, DQ164936, DQ164937, DQ164938, DQ164939, DQ164940, DQ164941, DQ164944, DQ164945, DQ164946, DQ164947, DQ164948,
DQ164949, DQ164950, DQ164951, DQ164952, DQ164953, DQ164954, DQ164955, DQ164956, DQ164957, DQ164958, DQ164959, DQ164960, DQ164961, DQ164962,
DQ164963, DQ164964, DQ164965, DQ164966, DQ164971, DQ164974, DQ164975, DQ164981, DQ164984, DQ164985, DQ164986, DQ164988, DQ164991, DQ164992,
DQ164993, DQ164994, DQ164995, DQ164996, DQ164997, DQ165022, DQ165023, DQ165024, DQ165025, DQ165026, DQ165027, DQ165028, DQ165029, DQ165030,
DQ165031, DQ165033, DQ165034, DQ165035, DQ165036, DQ165039, DQ165040, DQ165043, DQ165045, DQ165046, DQ165047, DQ165051, DQ165052, DQ165053,
DQ165054, DQ165055, DQ165056, DQ165057, DQ165058, DQ165059, DQ165060, DQ165061, DQ165062, DQ165063, DQ165064, DQ165065, DQ165068, DQ165070,
DQ248888, DQ296497, DQ296498, DQ296501, DQ296502, DQ296503, DQ296504, DQ296505, DQ296506, DQ296507, DQ296508, DQ296509, DQ296510, DQ296511,
DQ296512, DQ296513, DQ296514, DQ296515, DQ296516, DQ296517, DQ296518, DQ296519, DQ296522, DQ296523, DQ296524, DQ296525, DQ296526, DQ296527,
DQ296528, DQ296530, DQ296531, DQ372716, DQ478936, DQ478937, EF175732, EF457984, EF457985, EF457986, EF494498, EF494499, EF494500, EF494501,
EF494502, EF494503, EF494504, EF494505, EF494506, EF614457, EU109785, EU109786, EU139260, EU140964, EU244455, EU553846, EU553847, EU553848,
EU553849, EU667440, EU667441, EU667442, EU667443, EU667444, EU667445, EU667446, EU667447, EU667448, EU667449, EU667450, EU667451, EU667452,
EU667453, EU667454, FJ175661, FJ175662, FJ175663, FJ175664, FJ175665, FJ175666, FJ561310, FJ561311, FJ561312, FJ561313, FJ561314, FJ561315,
FJ561316, FJ561317, FJ561318, FJ561319, FJ561320, FJ561321, FJ798162, FJ798163, FJ798164, FJ798165, FJ798166, FJ798167, FJ798168, FJ798169,
FJ798170, FJ798171, FJ798172, FJ798173, FJ798174, FJ798175, FJ798176, FJ798177, FJ798178, FJ798179, FJ798180, FJ798181, FJ798182, FJ798183,
FJ798184, FJ798185, FJ798186, FJ798187, FJ798188, FJ798189, FJ798190, FJ798191, FJ798192, FJ798193, GQ292726, GQ292727, GQ292728, GQ292729,
GQ292730, GQ292731, GQ292732, GQ292733, GQ292734, GQ292735, GQ292736, GQ292737, GQ292738, GQ292739, GQ292740, GU082464, GU082465, GU082466,
GU082467, GU082468, GU082469, GU082470, GU082471, GU082472, GU082473, GU082474, GU082475, GU082476, GU082477, GU082478, GU082479, GU082480,
GU082481, GU125647, GU125648, GU125649, GU125650, GU384682, GU384683, GU384684, GU384685, GU582097, GU582098, GU582100, GU582101, GU582103,
GU582104, GU582106, GU582108, GU582111, GU582112, GU582113, GU582115, GU582116, GU582117, GU582119, GU582120, GU582121, GU582122, HM008917,
HM055494, HM055495, HM055497, HM055498, HM055499, HM055501, HM055505, HM055507, HM055508, HM055509, HM055510, HM143846, HM229661, HM561390,
HM561392, HM561393, HM561395, HM561396, HM561397, HM561398, HM561400, HM561402, HM561404, HM561405, HM561406, HM561407, HM561409, HM561410,
HM561411, HM561412, HM561414, HQ009509, HQ113232, HQ116171, HQ116172, HQ116173, HQ116174, HQ116175, HQ116176, HQ116177, HQ116178, HQ116179,
HQ116180, HQ116181, HQ116182, HQ116183, HQ116184, HQ116187, HQ116188, HQ116189, HQ116190, HQ116191, HQ116192, HQ116193, HQ116194, HQ116195,
HQ116196, HQ116197, HQ116198, HQ116199, HQ116200, HQ116201, HQ116202, HQ116203, HQ116204, HQ116205, HQ116206, HQ116207, HQ116208, HQ116209,
HQ116210, HQ116211, HQ116212, HQ116213, HQ116214, HQ116215, HQ116216, HQ116217, HQ116218, HQ116219, HQ116220, HQ116221, HQ116222, HQ116223,
HQ116224, HQ116225, HQ116226, HQ116227, HQ116228, HQ116229, HQ116230, HQ116231, HQ116232, HQ116233, HQ116234, HQ116235, HQ116236, HQ116237,
HQ116238, HQ116239, HQ116240, HQ116241, HQ116242, HQ116243, HQ116244, HQ116245, HQ116246, HQ116247, HQ116248, HQ116249, HQ116250, HQ116251,
HQ116252, HQ116253, HQ116254, HQ116255, HQ116256, HQ116257, HQ116258, HQ116259, HQ116260, HQ116261, HQ116262, HQ116263, HQ116264, HQ116265,
HQ116266, HQ116267, HQ116268, HQ116269, HQ116270, HQ116271, HQ116272, HQ116274, HQ116275, HQ116276, HQ116277, HQ116278, HQ116279, HQ116280,
HQ116281, HQ116282, HQ116283, HQ116284, HQ116285, HQ116286, HQ116288, HQ116289, HQ116290, HQ116291, HQ260713, HQ260714, HQ260715, HQ260716,
HQ260717, HQ260718, HQ260719, HQ260720, HQ268517, HQ268519, HQ268520, HQ268521, HQ268523, HQ268524, HQ268525, HQ268526, HQ412603, HQ439206,
HQ439207, HQ439208, HQ439209, HQ439210, HQ439211, HQ439212, HQ439213, HQ439214, HQ439215, HQ439216, HQ439217, HQ439218, HQ439219, HQ439220,

HQ439221, HQ439222, HQ439223, HQ439224, HQ439225, HQ439226, HQ439227, HQ439228, HQ439229, HQ439230, HQ439231, HQ439232, HQ439233, HQ439234,
HQ439235, HQ439236, HQ439237, HQ439238, HQ450645, HQ630676, HQ630677, HQ630678, HQ630679, HQ630680, HQ630681, HQ630682, HQ630683, HQ630684,
HQ630685, HQ630686, HQ630687, HQ630688, HQ630689, HQ630690, HQ630691, HQ630692, HQ630693, HQ630694, HQ630695, HQ630696, HQ632768, HQ632769,
HQ632770, HQ632771, HQ632772, HQ652078, HQ652079, HQ652080, HQ652081, HQ663879, JF288761, JF749846, JF749851, JF749852, JF749853, JF749855,
JF749856, JF749859, JF792356, JF837375, JF916985, JF916986, JF916987, JF937059, JF937060, JF937061, JF968122, JF968123, JF968124, JF968125,
JF968126, JF968127, JF968128, JF968129, JF968130, JF968131, JF968132, JF968133, JF968134, JF968135, JF968136, JF968137, JF968138, JF968139,
JF968140, JF968141, JF968142, JF968143, JF968144, JF968145, JF968146, JF968147, JF968148, JF968149, JF968150, JF968151, JF968152, JF968153,
JF968154, JF968155, JF968156, JF968157, JF968158, JF968159, JF968160, JF968161, JF968162, JF968163, JF968164, JF968165, JF968166, JF968167,
JF968168, JF968169, JF968170, JF968171, JF968172, JF968173, JF968174, JF968175, JF968176, JF968177, JF968178, JF968179, JF968180, JF968181,
JF968182, JF968183, JF968184, JF968185, JF968186, JF968187, JF968188, JF968189, JF968190, JF968191, JF968192, JF968193, JN676146, JN998085,
JN998086, JQ070301, JQ070302, JQ070303, JQ070304, JQ070305, JQ070306, JQ070307, JQ070308, JQ070309, JQ070310, JQ070311, JQ070312, JQ070313,
JQ070314, JQ070315, JQ070316, JQ070317, JQ070318, JQ070319, JQ070320, JQ070321, JQ070322, JQ070323, JQ070325, JQ070326, JQ070327, JQ070328,
JQ321837, JQ765581, JQ765582, JQ765583, JQ765584, JQ765585, JQ765586, JQ818555, JQ900581, JQ973889, JX040491, JX040492, JX040493, JX040494,
JX040495, JX040496, JX040497, JX040498, JX040499, JX040500, JX040501, JX070586, JX070587, JX070588, JX070589, JX070591, JX070592, JX070593,
JX070594, JX070595, JX070596, JX070597, JX070598, JX070599, JX070600, JX070601, JX070602, JX070603, JX070604, JX070605, JX070606, JX070607,
JX070608, JX070609, JX070610, JX070611, JX070612, JX070613, JX070614, JX070615, JX070616, JX070617, JX070618, JX070619, JX070620, JX070621,
JX070622, JX070623, JX070624, JX171676, JX171677, JX171678, JX171679, JX171680, KC438373, KC503937, KC506424, KC506425, KC506426, KC506427,
KC506428, KC506429, KC506430, KC506431, KC506432, KC506433, KC506434, KC506435, KC506436, KC506437, KC506438, KC506439, KC506440, KC506441,
KC506442, KC506443, KC506444, KC506445, KC506446, KC506447, KC506448, KC506449, KC506450, KC506451, KC506452, KC506453, KC506454, KC506455,
KC506456, KC506457, KC506458, KC506459, KC506460, KC506461, KC506462, KC506463, KC506464, KC506465, KC506466, KC506467, KC506468, KC506469,
KC506470, KC506471, KC506472, KC506473, KC506474, KC506475, KC506476, KC506477, KC506478, KC506479, KC506480, KC506481, KC506482, KC506483,
KC506484, KC506485, KC506486, KC506487, KC506488, KC506489, KC506490, KC506491, KC506492, KC506493, KC506494, KC506495, KC506496, KC506497,
KC506498, KC506499, KC506500, KC506501, KC506502, KC506503, KC506504, KC506505, KC506506, KC506507, KC506508, KC506509, KC506510, KC506511,
KC506512, KC506513, KC506514, KC506515, KC506516, KC506517, KC506518, KC506519, KC506520, KC506521, KC506522, KC506523, KC506524, KC506525,
KC506526, KC506527, KC506528, KC506529, KC506530, KC506531, KC506532, KC506533, KC506534, KC506535, KC506536, KC506537, KC506538, KC506539,
KC506540, KC506541, KC506542, KC506543, KC506544, KC506545, KC506546, KC506547, KC506548, KC506549, KC506550, KC506551, KC506552, KC506553,
KC506554, KC506555, KC506556, KC506557, KC506558, KC506559, KC506560, KC506561, KC506562, KC506563, KC506564, KC519631, KF112879, KF112880,
KF112881, KF112882, KF112885, KF112886, KF112887, KF112888, KF112889, KF321732, KF501486, KF501487, KF501488, KF694731, KF694732, KF694733,
KF694734, KF694735, KF694736, KF694737, KF694738, KF694739, KF694740, KF694741, KF694742, KF694743, KF694744, KF694745, KF694746, KF985189,
KJ175183, KJ175184, KJ175185, KJ206908, KJ206910, KJ427752, KJ606977, KJ606979, KJ606980, KJ606981, KJ606982, KJ606984, KJ825801, KJ825802,
KJ825803, KJ825804, KJ825805, KJ825806, KJ825807, KJ825808, KJ825809, KM243030, KM243034, KM243035, KM243036, KM243037, KM243038, KM243039,
KM243040, KM243041, KM243042, KM243043, KM243044, KM243045, KM243046, KM243047, KM243048, KM243049, KM243050, KM243051, KM243053, KM243054,
KM243055, KM243056, KM243057, KM243058, KM243059, KM243060, KM243061, KM243062, KM243063, KM243064, KM243065, KM243066, KM243067, KM243070,
KM243071, KM243072, KM243073, KM243074, KM243075, KM243076, KM243077, KM243078, KM243079, KM243080, KM243081, KM243082, KM243083, KM243084,
KM243085, KM243086, KM243087, KM243088, KM243089, KM243090, KM243091, KM243092, KM243093, KM243094, KM243095, KM243096, KM243097, KM243098,
KM243099, KM243100, KM243101, KM243102, KM243103, KM243104, KM243105, KM243107, KM243108, KM243109, KM243110, KM243111, KM243112, KM243113,
KM243114, KM243115, KM243116, KM243117, KM243118, KM243119, KM243120, KM243121, KM243122, KM243123, KM243124, KM243126, KM243127, KM243128,
KM243129, KM243130, KM243131, KM243132, KM243133, KM243134, KM243135, KM243136, KM243137, KM243138, KM243140, KM243141, KM243142, KM243143,
KM243144, KM243145, KM243146, KM243147, KM243148, KM243149, KM243150, KM243151, KM243152, KM243153, KM243154, KM243155, KM243156, KM243157,
KM243158, KM243159, KM243160, KM243161, KM243162, KM243163, KM243164, KM243165, KM243166, KM243167, KM243168, KM243169, KM243170, KM243171,
KM243172, KM264357, KM264358, KM264359, KM264360, KM264361, KM264362, KM264363, KM264364, KM268895, KM588384, KM588385, KM588386, KM588387,
KM588388, KM588389, KM588390, KM588391, KM588392, KM588393, KM588394, KM588395, KM588396, KM588397, KM588398, KM921813, KM921814, KM921815,
KM921816, KM921817, KM921818, KM921819, KM921820, KM921821, KM921822, KM921823, KM921824, KM921825, KM921826, KM921827, KM921828, KM921842,

KM921843, KM921844, KM921845, KM921846, KM921847, KM921848, KM921849, KM921850, KM921851, KM921852, KM921853, KM921854, KM921855, KM921856, KM921857, KM921858, KM921859, KM921860, KM921861, KM921862, KM921863, KM921864, KM921865, KM921866, KM921867, KM921868, KM921869, KM921870, KM921871, KM921872, KM921873, KM921874, KM921875, KM921877, KM921878, KP822942, KP822945, KP822946, KP822947, KP835578, KR149697, KR149698, KR149699, KR149700, KR149701, KR149702, KR149703, KR149704, KR149705, KR149706, KR149707, KR149713, KR149714, KR149715, KR149716, KR149717, KR149718, KR149719, KR401152, KR401153, KR401154, KR401155, KR401156, KR401157, KR401158, KR401159, KR401160, KT003716, KT037118, KT037119, KT037120, KT960948, KT982203, KU204893, KU204894, KU991728, KU991729, KU991730, KU991731, KU991732, KU991733, KU991734, KU991735, KU991736, KU991737, KX161429, KX162590, KX228159, KX228160, KX228161, KX534089, KX712091, KX944714, KX944715, KX944716, KX944717, KX944718, KX944719, KX944720, KX944721, KX944722, KX944723, KX944724, KX944725, KX944726, KX944727, KX944728, KX944729, KX944730, KX944731, KX944732, KX944733, KX944734, KX944735, KX944736, KY072818, KY077600, KY077601, KY077606, KY077607, KY077608, KY077609, KY077610, KY077612, KY077613, KY077621, KY086465, KY086466, KY091281, KY091282, KY091283, KY091284, KY091285, KY091286, KY091287, KY091288, KY234501, KY234502, KY322670, KY322671, KY322672, KY322673, KY322674, KY399460, KY399461, KY399463, KY399464, KY399465, KY399466, KY399467, KY399468, KY399469, KY399470, KY412559, KY412560, KY444644, KY444645, KY444646, KY444647, KY444648, KY444649, KY446903, KY449050, KY449051, KY449052, KY449053, KY449054, KY449055, KY492065, KY492066, KY492067, KY492068, KY492069, KY492070, KY492071, KY492072, KY492073, KY492074, KY492075, KY657269, KY659576, KY696707, KY696708, KY766147, KY766149, KY825719, KY825723, LC149617, LC149618, LC149619, LC149620, LC149621, LC149622, LC149623, LC149624, LC149625, LC149626, LC149627, LC149628, LC149629, LC149630, LC149631, LC149632, LC149633, LC149634, LC149635, LC149636, LC149637, LC149638, LC149639, LC149640, LC149641, LC149642, LC149643, LC149644, LC149645, LC149646, LC149647, LC149648, LC149649, LC149650, LC149651, LC149652, LC149653, LC149654, LC149655, LC149656, LC149657, LC149658, LC149659, LC149660, LC149661, LC149662, LC149663, LC149664, LC149665, LC149666, LC149667, LC149668, LC149669, LC149670, LC149671, LC149672, LC149673, LC149674, LC149675, LC149676, LC149677, LC149678, LC149679, LC149680, LC149681, LC149682, LC149683, LC149684, LC149685, LC149686, LC149687, LC149688, LC149689, LC149690, LC149691, LC149692, LC149693, LC149694, LC149695, LC149696, LC149697, LC149698, LC149699, LC149700, LC149701, LC149702, LC149703, LC149704, LC149705, LC149706, LC149707, LC149708, LC149709, LC149710, LC149711, LC149712, LC149713, LC149714, LC149715, LC149716, LC149717, LC149718, LC149719, LC149720, LC320038, LC438819, LC438820, LC438821, LC438822, LC438823, LC439252, LC439253, LC439254, LC595604, MF143572, MF143573, MF143574, MF143575, MF143576, MF143577, MF143578, MF374987, MF461724, MF947123, MF947124, MF947125, MF947126, MF947127, MF947128, MF947129, MF947130, MF947131, MF947132, MF947134, MF947135, MF947136, MF947137, MF947138, MF947139, MF947140, MF947141, MF947142, MF947143, MF947453, MF947454, MF947455, MF947456, MF947457, MF947458, MF947459, MF947460, MF947461, MF947462, MF947463, MF947464, MF947465, MF947466, MF947467, MF947468, MF947469, MF947470, MF947471, MF947472, MF947473, MF947474, MF947475, MF947476, MF947477, MF947478, MF947479, MF947480, MF947481, MF947482, MF947483, MF947484, MF947485, MF947486, MF947487, MF947488, MF947489, MF947490, MF947491, MF947492, MF947493, MG257781, MG257782, MG257783, MG257784, MG257785, MG257786, MG372730, MG840803, MG893512, MG893513, MG893514, MG893515, MG893516, MG893517, MG893518, MG893519, MG893520, MG893521, MG893522, MG893523, MG893524, MG893525, MG893526, MG893527, MG893528, MG893529, MG893530, MG893531, MG893532, MG893533, MG893534, MG893535, MG893536, MG893537, MG893538, MG893539, MG893540, MG893541, MG893542, MG893543, MG893544, MG893545, MG893546, MG893547, MG893548, MG893549, MG893550, MG893551, MG893552, MG972465, MG972466, MG972467, MG972468, MG972469, MG972470, MG972471, MG972472, MG972473, MG972474, MG972475, MG972476, MG972477, MG972478, MG972479, MG972480, MG972481, MG972482, MG972483, MG972484, MG972485, MG972486, MG972487, MG972488, MG972489, MG972516, MG972517, MG972518, MG972519, MG972520, MG972521, MG972522, MG972523, MG972524, MG972525, MG972526, MG972527, MG972528, MG972529, MG972530, MG972531, MG972532, MG972533, MG972534, MG972535, MG972536, MG972537, MG972538, MG972539, MG972540, MG972541, MG972543, MG972544, MG972545, MG972546, MG972547, MG972548, MG972549, MG972550, MG972551, MG972552, MG972553, MG972554, MG972555, MG972556, MG972557, MG972558, MG972559, MG972560, MG972561, MG972562, MG972563, MG972564, MG972565, MG972566, MG972567, MG972568, MG972569, MG972570, MG972571, MG972572, MG972573, MG972574, MG972575, MG972576, MG972577, MG972578, MG972579, MG972580, MG972581, MG972582, MG972586, MG972587, MG972588, MG972589, MG972590, MG972591, MG972592, MG972593, MG972594, MG972595, MG972596, MG972597, MG972598, MG972599, MG972600, MG972601, MG972602, MG972605, MG972606, MG972607, MG972608, MG972609, MG972610, MG972611, MG972612, MG972613, MG972614, MG972615, MG972616, MG972617, MG972618, MG972619, MG983684, MG983685, MG983686, MG983687, MG983688, MG983689, MG983690, MG983691, MG983692, MG983693, MG983703, MG983704, MG983705, MG983706, MG983707, MG983708, MG983709, MG983710, MG983711, MG983712, MG983713, MG983714, MG983715, MG983716, MG983717, MG983718, MG983719, MG983721, MG983722, MG983723, MG983724, MG983725, MG983726, MG983727, MG983728, MG983729, MG983730, MG983731, MG983732, MG983733, MG983734, MG983736, MG983737, MG983738, MG983739, MG983740, MG983741, MH053353, MH784403, MH784404, MH784405, MH791315, MH791316, MH791317, MH791318, MH807443, MH845413, MH891503, MK071699,

MK088170, MK292141, MK292142, MK292143, MK292144, MK342179, MK342180, MK342181, MK342182, MK342183, MK342184, MK342185, MK342186, MK342187, MK342188, MK342190, MK390887, MK390888, MK390889, MK390890, MK390891, MK390892, MK390895, MK390896, MK390897, MK390898, MK390899, MK390900, MK390901, MK390902, MK390903, MK390904, MK390905, MK390906, MK390907, MK390908, MK390909, MK390910, MK390911, MK390912, MK390913, MK390914, MK390915, MK390916, MK390917, MK390918, MK390920, MK390921, MK390922, MK390923, MK390924, MK390925, MK390926, MK390927, MK390928, MK390929, MK390930, MK390931, MK390932, MK390933, MK390934, MK390935, MK390936, MK390937, MK390938, MK390939, MK390940, MK511175, MK511176, MK511177, MK511178, MK511179, MK511180, MK511181, MK511182, MK511183, MK511184, MK511185, MK511186, MK511187, MK511188, MK511189, MK511190, MK934700, MK934701, MK934702, MK934703, MK934704, MK934706, MK934707, MN056182, MN062577, MN062578, MN062579, MN062580, MN062581, MN062582, MN062583, MN062584, MN062585, MN095354, MN095355, MN095356, MN095357, MN095358, MN095359, MN095360, MN095361, MN095362, MN095363, MN095364, MN095365, MN095366, MN095367, MN095368, MN095369, MN095370, MN095371, MN103289, MN250314, MN250315, MN250316, MN250317, MN250318, MN276040, MN276041, MN276044, MN276046, MN379784, MN379785, MN379786, MN379787, MN379788, MN379789, MN379790, MN379791, MN379792, MN379793, MN379794, MN379795, MN379796, MN379797, MN379798, MN379799, MN379800, MN379801, MN379802, MN379803, MN379804, MN379805, MN379806, MN379807, MN379808, MN379809, MN379810, MN379811, MN379812, MN379813, MN379814, MN379815, MN379816, MN379817, MN379818, MN379819, MN379820, MN379821, MN379822, MN379823, MN389541, MN547618, MN953619, MN953620, MN966686, MN983152, MN983153, MN983154, MN983155, MN983156, MN983158, MT018548, MT018549, MT018550, MT018551, MT113939, MT113940, MT113941, MT164527, MT164528, MT164529, MT180749, MT180750, MT180752, MT180758, MT180760, MT180762, MT185941, MT215164, MT215165, MT276847, MT276848, MT276849, MT276850, MT316587, MT316590, MT442814, MT442815, MT442816, MT442817, MT442818, MT442819, MT442820, MT442821, MT442822, MT442823, MT442824, MT442825, MT442826, MT442827, MT442828, MT442829, MT442830, MT442831, MT442832, MT442833, MT442834, MT442835, MT442836, MT442837, MT442838, MT442839, MT442840, MT442841, MT442842, MT442843, MT442844, MT442845, MT442846, MT442847, MT442848, MT442849, MT442850, MT442851, MT442852, MT442853, MT442854, MT442855, MT442856, MT442857, MT442858, MT442859, MT442860, MT442861, MT442862, MT442863, MT442864, MT442865, MT442866, MT442867, MT442868, MT442869, MT442870, MT442871, MT442872, MT442873, MT442874, MT442875, MT442876, MT442877, MT442878, MT442879, MT442880, MT442881, MT442882, MT442883, MT442884, MT442885, MT442886, MT442887, MT442888, MT442889, MT442890, MT442891, MT442892, MT442893, MT442894, MT442895, MT442896, MT442897, MT442898, MT442899, MT442900, MT442901, MT442902, MT442903, MT442904, MT442905, MT442906, MT442907, MT442908, MT442909, MT442910, MT442911, MT442912, MT442913, MT442914, MT442915, MT442916, MT442917, MT442918, MT442919, MT442920, MT442921, MT442922, MT442923, MT442924, MT442925, MT442926, MT442927, MT442928, MT442929, MT442930, MT442931, MT442932, MT442933, MT442934, MT442935, MT442936, MT442937, MT442938, MT442939, MT442940, MT442941, MT442942, MT442943, MT442944, MT442945, MT442946, MT442947, MT442948, MT442949, MT442950, MT442951, MT442952, MT442953, MT442954, MT442955, MT442956, MT442957, MT442958, MT442959, MT442960, MT442961, MT442962, MT442963, MT442964, MT442965, MT442966, MT442967, MT442968, MT442969, MT442970, MT442971, MT442972, MT442973, MT442974, MT442975, MT442976, MT442977, MT442978, MT442979, MT442980, MT442981, MT442982, MT442983, MT442984, MT442985, MT442986, MT442987, MT442988, MT442989, MT442990, MT442991, MT442992, MT442993, MT442994, MT442995, MT442996, MT442997, MT442998, MT442999, MT443000, MT443001, MT443002, MT443003, MT443004, MT443005, MT443006, MT443007, MT443008, MT443009, MT443010, MT443011, MT443012, MT443013, MT443014, MT443015, MT443016, MT443017, MT443018, MT443019, MT443020, MT443021, MT443022, MT443023, MT443024, MT443025, MT443026, MT443027, MT443028, MT443029, MT443030, MT443031, MT443032, MT443033, MT443034, MT443035, MT443036, MT443037, MT443038, MT443039, MT443040, MT443041, MT443042, MT443043, MT443044, MT443045, MT443046, MT443047, MT443048, MT443049, MT443050, MT443051, MT443052, MT443053, MT443054, MT443055, MT443056, MT443057, MT443058, MT443060, MT443061, MT443062, MT443081, MT443082, MT443083, MT443084, MT443085, MT443086, MT443087, MT443088, MT443089, MT443090, MT443091, MT443092, MT443093, MT443094, MT443095, MT443096, MT443097, MT443098, MT443099, MT443100, MT443101, MT443102, MT443103, MT443104, MT443105, MT443106, MT443107, MT443108, MT443109, MT443110, MT443111, MT443112, MT443113, MT443114, MT443115, MT443116, MT443117, MT443118, MT443119, MT443120, MT443121, MT443122, MT443123, MT443124, MT443125, MT443126, MT443127, MT443128, MT443129, MT443130, MT443131, MT443132, MT443133, MT443134, MT443135, MT443136, MT443137, MT443138, MT443139, MT443140, MT443141, MT443142, MT443143, MT443144, MT443145, MT443146, MT443147, MT443148, MT443149, MT443150, MT443151, MT443152, MT443153, MT443154, MT443155, MT443156, MT443157, MT443158, MT443159, MT443160, MT443161, MT443162, MT443163, MT443164, MT443165, MT443166, MT443167, MT443168, MT443169, MT443170, MT443171, MT443172, MT443173, MT443174, MT443175, MT443176, MT443177, MT443178, MT443179, MT443180, MT443181, MT443182, MT443183, MT443184, MT443185, MT443186, MT443187, MT443188, MT443189, MT443190, MT443191, MT443192, MT443193, MT443194, MT443195, MT443196, MT443197, MT443198, MT443199, MT443200, MT443201, MT443202, MT443203, MT443204, MT443205, MT443206, MT443207, MT443208, MT443209, MT443210, MT443211, MT443212, MT443213, MT443214, MT443215, MT443216, MT443217, MT443218, MT443219, MT443220, MT443221, MT443222, MT443223, MT443224, MT443225, MT443226, MT443227, MT443228, MT443229, MT443230,

MT443231, MT443232, MT443233, MT443234, MT443235, MT443236, MT443237, MT443238, MT443239, MT443240, MT443241, MT443242, MT443243, MT443244,
MT443245, MT443246, MT443247, MT443248, MT443249, MT443250, MT443251, MT443252, MT443253, MT443254, MT443255, MT443256, MT443257, MT443258,
MT443259, MT443260, MT443261, MT443262, MT443263, MT443264, MT443265, MT443266, MT443267, MT443268, MT443269, MT443270, MT443271, MT443272,
MT443273, MT443274, MT443275, MT443276, MT443277, MT443278, MT443279, MT443280, MT443281, MT443282, MT443283, MT443284, MT443285, MT443286,
MT443287, MT443288, MT443289, MT443290, MT443291, MT443292, MT443293, MT443294, MT443295, MT443296, MT443297, MT443298, MT443299, MT443300,
MT443301, MT443302, MT443303, MT443304, MT443305, MT443306, MT443307, MT443308, MT443309, MT443310, MT443311, MT443312, MT443313, MT443314,
MT443315, MT443316, MT443317, MT443318, MT443319, MT443320, MT443321, MT443322, MT443323, MT443324, MT443325, MT443326, MT443327, MT443328,
MT443329, MT443330, MT443331, MT443332, MT443333, MT443334, MT443335, MT443336, MT443337, MT443338, MT443339, MT443340, MT443341, MT443342,
MT443343, MT443344, MT443345, MT443346, MT443347, MT443348, MT443349, MT443350, MT443351, MT443352, MT443353, MT443354, MT443355, MT443356,
MT443357, MT443358, MT443359, MT443360, MT443361, MT443362, MT443363, MT443364, MT443365, MT443366, MT443367, MT443368, MT443369, MT443370,
MT443371, MT443372, MT443373, MT443374, MT443375, MT443376, MT443377, MT443378, MT443379, MT443380, MT443381, MT443382, MT443383, MT443384,
MT443385, MT443386, MT443387, MT443388, MT443389, MT443390, MT443391, MT443392, MT443393, MT443394, MT443395, MT443396, MT443397, MT443398,
MT443399, MT443400, MT443401, MT443402, MT443403, MT443404, MT443405, MT443406, MT443407, MT443408, MT443409, MT443410, MT443411, MT443412,
MT443413, MT443414, MT443415, MT443416, MT443417, MT443418, MT443419, MT443420, MT443421, MT443422, MT443423, MT443424, MT443425, MT443426,
MT443427, MT443428, MT443429, MT443430, MT443431, MT443432, MT443433, MT443434, MT443435, MT443436, MT443437, MT443438, MT443439, MT443440,
MT443441, MT443442, MT443443, MT443444, MT443445, MT443446, MT443447, MT443448, MT443449, MT443450, MT443451, MT443452, MT443453, MT443454,
MT443455, MT443456, MT443457, MT443458, MT443459, MT443460, MT443461, MT443462, MT443463, MT443464, MT443465, MT443466, MT443467, MT443468,
MT443469, MT443470, MT443471, MT443472, MT443473, MT443474, MT443475, MT443476, MT443477, MT443478, MT443479, MT443480, MT443481, MT443482,
MT443483, MT443484, MT443485, MT443486, MT443487, MT443488, MT443489, MT443490, MT443491, MT443492, MT443493, MT443494, MT443495, MT443496,
MT443497, MT443498, MT443499, MT443500, MT443501, MT443502, MT443503, MT443504, MT443505, MT443506, MT443507, MT443508, MT443509, MT443510,
MT443511, MT443512, MT443513, MT443514, MT443515, MT443516, MT443517, MT443518, MT443519, MT443535, MT443536, MT443537, MT443538, MT443539,
MT443540, MT443541, MT443542, MT443543, MT443544, MT443545, MT443546, MT443547, MT443548, MT443549, MT443550, MT443551, MT443552, MT443553,
MT443554, MT443555, MT443556, MT443557, MT443558, MT443559, MT443560, MT443561, MT443562, MT443563, MT443564, MT443565, MT443566, MT443567,
MT443568, MT443569, MT443570, MT443571, MT443572, MT443573, MT443574, MT443575, MT443576, MT443577, MT443578, MT443579, MT443580, MT443581,
MT443582, MT443583, MT443584, MT443585, MT443586, MT443587, MT443588, MT443589, MT443590, MT443591, MT443592, MT443593, MT443594, MT443595,
MT443596, MT443597, MT443598, MT443599, MT443600, MT443601, MT443602, MT443603, MT443604, MT443605, MT443606, MT443607, MT443608, MT443609,
MT443610, MT443611, MT443612, MT443613, MT443614, MT443615, MT443616, MT443617, MT443618, MT443619, MT443620, MT443621, MT443622, MT443623,
MT443624, MT443625, MT443626, MT443627, MT443628, MT443629, MT443630, MT443631, MT443632, MT443633, MT443634, MT443635, MT443636, MT443637,
MT443638, MT443639, MT443640, MT443641, MT443642, MT443643, MT443644, MT443645, MT443646, MT443647, MT443648, MT443649, MT443650, MT443651,
MT443652, MT443653, MT443654, MT443655, MT443656, MT443657, MT443658, MT443659, MT443660, MT443661, MT443662, MT443663, MT443664, MT443665,
MT443666, MT443667, MT443668, MT443669, MT443670, MT443671, MT443672, MT443673, MT443674, MT443675, MT443676, MT443677, MT443678, MT443679,
MT443680, MT443681, MT443682, MT443683, MT443684, MT443685, MT443686, MT443687, MT443688, MT443689, MT443690, MT443691, MT443692, MT443693,
MT443694, MT443695, MT443696, MT443697, MT443698, MT443699, MT443700, MT443701, MT443702, MT443703, MT443704, MT443705, MT443706, MT443707,
MT443708, MT443709, MT443710, MT443711, MT443712, MT443713, MT443714, MT443715, MT443716, MT443717, MT443718, MT443719, MT443720, MT443721,
MT443722, MT443723, MT443724, MT443725, MT443726, MT443727, MT443728, MT443729, MT443730, MT443731, MT443732, MT443733, MT443734, MT443735,
MT443736, MT443737, MT443738, MT443739, MT443740, MT443741, MT443742, MT443743, MT443744, MT443745, MT443746, MT443747, MT443748, MT443749,
MT443750, MT443751, MT443752, MT443753, MT443754, MT443755, MT443756, MT443757, MT443758, MT443759, MT443760, MT443761, MT443762, MT443763,
MT443764, MT443765, MT443766, MT443767, MT443768, MT443769, MT443770, MT443771, MT443772, MT443773, MT443774, MT443775, MT443776, MT443777,
MT443778, MT443779, MT443780, MT443781, MT443782, MT443783, MT443784, MT443785, MT443786, MT443787, MT443788, MT443789, MT443790, MT443791,
MT443792, MT443793, MT443794, MT443795, MT443796, MT443797, MT443798, MT443799, MT443800, MT443801, MT443802, MT443803, MT443804, MT443805,
MT443806, MT443807, MT443808, MT443809, MT443810, MT443811, MT443812, MT443813, MT443814, MT443815, MT443816, MT443817, MT443818, MT443819,
MT443820, MT443821, MT443822, MT443823, MT443824, MT443825, MT443826, MT443827, MT443828, MT443829, MT443830, MT443831, MT443832, MT443833,
MT443834, MT443835, MT443836, MT443837, MT443838, MT443839, MT443840, MT443841, MT443842, MT443843, MT443844, MT443845, MT443846, MT443847,

MT443848, MT443849, MT443850, MT443851, MT443852, MT443853, MT443854, MT443855, MT443856, MT443857, MT443858, MT443859, MT443860, MT443861, MT443862, MT443863, MT443864, MT443865, MT443866, MT443867, MT443868, MT443869, MT443870, MT443871, MT443872, MT443873, MT443874, MT443875, MT443876, MT443877, MT443878, MT443879, MT443880, MT443881, MT443882, MT443883, MT443884, MT443885, MT443886, MT443887, MT443888, MT443889, MT443890, MT443891, MT909571, MT909572, MT909573, MT909574, MT909575, MT909576, MT909577, MT909578, MT909579, MT909580, MT909581, MT909582, MT909583, MT909584, MT909585, MT909586, MT909587, MT909588, MT909589, MT909590, MT909591, MT909592, MT909593, MT909594, MT909595, MT909596, MT909597, MT909598, MT909599, MT909600, MT909601, MT909602, MT909603, MT909604, MT909605, MT909606, MT909607, MT909608, MT909609, MT909610, MT909611, MT909612, MT909613, MT909614, MT909615, MT909616, MT909617, MT909618, MT909619, MT909620, MT909621, MT909622, MT909623, MT909624, MT909625, MT909626, MT909627, MT909628, MT909629, MT909630, MT909631, MT909632, MT909633, MT909634, MT909635, MT909636, MT909637, MT909638, MT909639, MT909640, MT909641, MT909642, MT909643, MT909644, MT909645, MT909646, MT909647, MT909648, MT909649, MT909650, MT909651, MT909652, MT909653, MT909654, MT909655, MT909656, MT909657, MT909658, MT909659, MT909660, MT909661, MT909662, MT909663, MT909664, MT909665, MT909666, MT909667, MT909668, MT909669, MT909670, MT909671, MT909672, MT909673, MT909674, MT909675, MT909676, MT909677, MT909678, MT909679, MT909680, MT909681, MT909682, MT909683, MT909684, MT909685, MT909686, MT909687, MT909688, MT909689, MT909690, MT909691, MT909692, MT909693, MT909694, MT909695, MT909696, MT909697, MT909698, MT909699, MT909700, MT909701, MT909702, MT909703, MT909704, MT909705, MT909706, MT909707, MT909708, MT909709, MT909710, MT909711, MT909712, MT909713, MT909714, MT909715, MT909716, MT909717, MT909718, MT909719, MT909720, MT909721, MT909722, MT909723, MT909724, MT909725, MT909726, MT909727, MT909728, MT909729, MT909730, MT909731, MT909732, MT909733, MT909734, MT909735, MT909736, MT909737, MT909738, MT909739, MT909740, MT909741, MT909742, MT909743, MT909744, MT909745, MT909746, MT909747, MT909748, MT909749, MT909750, MT909751, MT909752, MT909753, MT909754, MT909755, MT909756, MT909757, MT909758, MT909759, MT909760, MT909761, MT909762, MT909763, MT909764, MT909765, MT909766, MT909767, MT909768, MT909769, MT909770, MT909771, MT909772, MT909773, MT909774, MT909775, MT909776, MT909777, MT909778, MT909779, MT909780, MT909781, MT909782, MT918973, MT918974, MT918975, MT918976, MT918977, MT918978, MT918979, MT918980, MT918981, MT918982, MT918983, MT918984, MT918985, MT918986, MT918987, MT918988, MT918989, MT918990, MT918991, MT918992, MT918993, MT918994, MT918995, MT918996, MT918997, MT918998, MT918999, MT919000, MT919001, MT919002, MT919003, MT919004, MT919005, MT919006, MT919007, MT919008, MT919009, MT919010, MT919011, MT919012, MT919013, MT919014, MT919015, MT919016, MT919017, MT936089, MT936090, MT936091, MT936092, MT936093, MT936094, MT936095, MT936096, MT936097, MT936098, MT936099, MT936100, MT936101, MT936102, MT936103, MT936104, MT936105, MT936106, MT936107, MT936108, MT936109, MT936110, MT936111, MT936112, MT936113, MT936114, MT936115, MT936116, MT936117, MT944980, MT944981, MT944982, MT944983, MW085845,MW085846, MW085847,MW085848,MW085849,MW085850,MW085851,MW085852,MW085853,MW085854,MW085855,MW085856,MW085857,MW085858,MW085859,MW085860, MW085861,MW085862,MW085863,MW085864,MW085865,MW085866,MW085867,MW085868,MW085869,MW085870,MW085871,MW085873,MW085875,MW085876, MW085877,MW085878,MW085879,MW085880,MW085881,MW085882,MW085883,MW085884,MW085885,MW085886,MW085887,MW085888,MW085889,MW085890, MW085891,MW085893,MW085894,MW085895,MW085896,MW085906,MW085907,MW085908,MW085909,MW085910,MW085911,MW085912,MW085913,MW524745, MW524748,MW558028,MW558029,MW558030,MW558031,MW558032,MW558033,MW558034,MW558035,MW558036,MW558037,MW558038,MW558039,MW558040, MW558041,MW558042,MW558043,MW558044,MW558045,MW558046,MW712670,MW712671,MW712672,MW712673,MW712674,MW712675,MW712676,MW712677, MZ546619, MZ546621, OK019689, OK359042, OK359043, OK359044, OK359045, OK422489, OK422490, OK422491, OM221277, OM221278, OM221279, OM221280, OM221281, OM221293, OM221294, OM221295, OM221296, OM221297, OM221298, OM221299, OM221300, OM221301, OM221302, OM221303, OM221305, OM221306, OM221307, OM221308, OM221309, OM221310, OM221311, OM221312, OM221313, OM221314, OM221315, OM221316, OM221317, OM221318, OM221319, OM221320, OM221321, OM221322, OM221323, OM221324, OM221325, OM221326, OM221327, OM221328, OM221329, OM221330, OM221331, OM221332, OM221333, OM221334, OM221335, OM221336, OM221337, OM221338, OM221339, OM221340, OM221341, OM221342, OM221343, OM221344, OM221345, OM221346, OM221347, OM221348, OM221349, OM221350, OM221351, OM456128, OM456129, OM456130, OM456131, OM456132, OM456133, OM456134, OM456135, OM456136, OM456137, OM456138, OM456139, OM456140, OM456141, OM456142, OM456143, OM456144, OM456145, OM456146, OM456147, OM456148, OM456149, OM456150
